# Political polarization on the move: Analyzing geographical mobility between counties in the U.S.

**DOI:** 10.1371/journal.pone.0339333

**Published:** 2026-01-28

**Authors:** Zhengyi Liang, Jaeho Cho

**Affiliations:** Department of Communication, University of California, Davis, California, United States of America; University of Connecticut, UNITED STATES OF AMERICA

## Abstract

Research on geographical polarization typically focuses on residential segregation by partisanship, where individuals with similar political affiliations cluster in the neighborhoods. Our study extends this line of research beyond residential spaces by investigating the influence of political leaning on geographical mobility in non-residential, activity spaces. Specifically, by analyzing human mobility patterns between counties, we explore the effects of political leaning and the strength of political leaning on the preference for politically similar locations. We find that while political leaning does not significantly impact mobility preferences, the strength of political leaning does, even after controlling for economic, demographic, and other contextual factors. Individuals traveling from counties with a strong political leaning are more likely to visit politically congruent destinations. This finding suggests that everyday travel may align with political preferences in counties with politically homogeneous populations. Our study highlights a new research avenue for research on geographical polarization by focusing on human mobility, providing unique insights into how political divisions influence spatial segregation.

## Introduction

Political polarization, which originated in the public domain, has infiltrated the private sphere of Americans [1,2]. A significant development of this trend is its geographical manifestation. Partisans increasingly cluster in residential areas with like-minded neighbors [3]. Such political segregation in physical spaces creates partisan bubbles and limits opportunities for exposure to and engagement with cross-cutting political views, which in turn deepens the ideological divide [4]. In the context of residential segregation, we further investigate whether human mobility reinforces existing geographical divisions by aligning with political preferences or whether diverse geographical interactions and communication help to bridge the political divide. Specifically, by analyzing human mobility patterns between counties, we examine the impacts of political leaning and the strength of political leaning on the tendency to favor politically similar destinations.

A significant body of literature has focused on spatial segregation by race and income [5,6], reflecting social stratification within society. Research has also explored segregation and social mixing along other dimensions, including gender and ethnicity [7–10]. More recently, scholars have discovered residential segregation driven by partisanship [3], motivated by growing dislike and distrust towards those from opposing political camps [11]. However, we argue that studying residential choice alone does not fully capture the pattern of geographical polarization and the role of political leaning in shaping spatial segregation. Physical areas typically consist of both static residential spaces and dynamic activity spaces [12], highlighting that people make decisions not only about where to live but also about where to go. This suggests that existing research, which has primarily focused on the residential divide along partisan lines, offers a limited understanding of partisan geographical polarization.

Our study addresses this gap by investigating how political leaning influences geographical mobility. By examining these patterns, we extend the concept of geographical polarization beyond residential neighborhoods to encompass the dynamic spaces individuals traverse in daily life. Political polarization, we argue, may have spilled over into another private dimension of everyday life: travel. This underexplored phenomenon provides fertile ground for new insights and opportunities for intervention [12]. Furthermore, our study has practical implications for designing contextual interventions aimed at curbing political polarization. Understanding whether and how individuals’ physical movements are shaped by political leanings sheds light on the conditions under which diverse geographical communication and interactions are more likely to occur. Such insights can inform the development of targeted interventions that encourage cross-cutting contact and foster opportunities to bridge political divides.

In the following sections, we start by reviewing the literature on geographical polarization and previous research exploring the relationship between human mobility, political leaning and its strength. We then conduct a computational analysis of phone tracking data and voting records to investigate how political leaning and its strength influence mobility preferences for politically similar locations. Finally, we conclude this paper by discussing the findings and their implications.

### Geographical polarization

Geographical segregation, driven by political polarization, refers to the phenomenon that areas are increasingly dominated by those from one political party across various geographical scales [13]. A growing body of literature has examined geographical segregation in residential areas across the U.S., highlighting the limited exposure to opposing political views in residential environments [3,14,15]. The literature on geographical segregation serves as the background of the present study. First, this line of research offers solid evidence to justify the merits of examining political polarization through a geographical perspective. Polarization occurs not only at the individual level but also at broader societal scales. Second, research reveals the tendency of partisans to cluster in ideologically homogeneous residential districts with like-minded neighbors [3]. Building on this, we further investigate whether individuals extend these patterns of ideological alignment into their mobility behavior, conforming to existing divisions by traveling to like-minded locations.

### Political leaning and geographical mobility

Partisan and ideological identities are regarded as stable social identities [16,17]. Social identity develops through social interactions, as individuals express their sense of belonging and display identity-relevant behaviors through communicating with others [18]. According to social identity theory (SIT), identification with a group not only defines who we are but also guides what we do and how we feel [19]. Thus, political identity has the potential to influence our behaviors and emotions [20].

An emerging body of research explores the relationship between partisanship and mobility [21–23], defined as the physical movement of individuals between locations, whether permanent (e.g., migration) or temporary (e.g., commuting) [24]. Our study extends this strand of research by examining the association between political leaning and mobility preference for traveling to politically similar locations. Drawing on SIT, political leaning may shape mobility patterns by encouraging individuals to seek out like-minded others and to travel to destinations that align with familiar lifestyles. Specifically, this relationship may be driven by political homophily—the tendency to connect with similar others [25]. Traveling to politically congruent destinations offers opportunities for interaction with like-minded individuals, fostering social comfort and cohesion. It may also reflect lifestyle preferences, as ideological divisions increasingly shape everyday choices in leisure, culture, and consumption [1,2,26]. Visiting politically similar areas thus enables individuals to sustain familiar routines and activities even while away from home.

This study examines the relationship between political leaning and mobility preferences for traveling to politically similar locations. We expect that individuals from conservative areas show a stronger tendency toward politically congruent travel, which is grounded in the framework of ingroup–outgroup differentiation and social influence. Republicans are more likely than Democrats to attribute negative traits to political opponents [27], and the relatively homogeneous and rural environments of many Republican-leaning counties may intensify partisan antipathy by limiting cross-partisan contact. In the absence of direct interaction, residents may rely on partisan media portrayals of “liberal” places, reinforcing reluctance to travel there. We therefore hypothesize:

H1: Political leaning is associated with mobility preferences for traveling to politically similar locations. Specifically, individuals traveling from conservative counties are more likely to visit politically similar destinations than those traveling from liberal counties.

### Political leaning strength and geographical mobility

The effect of political leaning on geographical mobility may vary depending on the context. In this study, we examine whether the influence of partisanship on mobility preferences is more pronounced among individuals with strong political leanings. Political leaning strength refers to the extent to which individuals identify themselves as strongly conservative or strongly liberal [28]. Its effects are most pronounced among those who firmly identify with their political party [29]. Strong partisans, in particular, exhibit heightened affective polarization, displaying stronger favoritism toward in-group members and greater hostility toward out-group members [11,20]. Thus, their intense political identity, conservative or liberal, drives them to seek alignment with like-minded individuals and to differentiate themselves from opposing groups [30].

We predict that the relationship between partisanship and mobility preferences is particularly strong among political stalwarts, whether conservative or liberal. Strong partisans on both sides may possess a higher level of motivation to travel to politically similar areas, where they can connect with like-minded individuals and immerse themselves in social demographic environments and lifestyles aligned with their political beliefs. Based on this reasoning, we propose the following hypothesis:

H2: Political leaning strength is positively associated with mobility preference for traveling to politically similar locations.

## Materials and methods

### Data

This study utilizes two datasets. The first dataset is based on county-level presidential election returns from the 2020 presidential election, released by the MIT Media Lab and accessible at https://dataverse.harvard.edu/dataset.xhtml?persistentId=doi:10.7910/DVN/VOQCHQ. These voting records allow us to quantify the political leanings of counties in the United States, a measure that will be elaborated upon in the subsequent section. The second dataset is phone tracking data that document trips made by individuals at the census tract level between November 2018 and November 2019. The anonymized phone data, collected by SafeGraph from over 40 million U.S. smartphones, was retrieved from Brazil [31]. To align the phone tracking data with the election data, we aggregated it at the county level. Following this aggregation, the analysis includes a total of 471 counties.

### Measurements

#### Political leaning.

We calculated the political leanings L of counties using presidential election results. Researchers commonly scale the political leanings of states based on election outcomes [1,32], and we adopted this approach at the county level. Specifically, L was measured as the difference between Republican votes and Democratic votes in the 2020 general election. The formula is presented below.


$$L\;=\;P_{i}-P_{j}$$

Where $P_{i}$ denotes the percentage of Republican votes, calculated as the number of Republican votes divided by the total votes, while $P_{j}$ represents the percentage of Democratic votes, calculated as the number of Democratic votes divided by the total votes. Note that total votes include Republican, Democratic, and other votes.

#### Political leaning strength.

In survey research, partisanship strength is often measured by folding the scale of partisanship onto itself [33], a method also known as a folded measure. Following this approach, we created a measure of political leaning strength by folding the political leaning scale onto itself to create a scale ranging from 0 to 1. Higher values indicate stronger political leanings within a county, while lower values suggest weaker political leanings. Additional details are provided in Figs 2a–c.

#### Traveling polarization index.

We constructed a measurement, traveling polarization index, to capture the extent to which individuals prefer traveling to politically similar counties over politically opposing counties. Conceptually, mobility preference is an attitudinal construct; however, in this study we operationalize it behaviorally through observed trips between politically different locations. This approach aligns with SIT, which predicts that political identity shapes not only attitudes but also behaviors. Our operationalization suggests that how people move signals their true preferences for mobility options. This revealed-preference approach avoids self-report bias in surveys, improving the validity of the measurement. Also, it allows us to analyze more populations through large-scale travel data that captures more heterogeneity and nuances than surveys. Such operationalization is common in mobility and urban studies, where actual trips are used to infer underlying preferences [35–37].

Based on the political leaning measurement, we categorized counties into three groups. Counties with L <−3% were classified as liberal or blue, those with −3% ≤ L ≤ 3% were considered centrist or purple, and counties with L > 3% were identified as conservative or red. By setting the cutoff at ±3%, we underscore that purple counties represent electorates essentially split down the middle. This provides a clearer conceptual separation: blue and red counties exhibit an explicit partisan preference, whereas purple counties do not. Under our definition, a purple county’s political leaning is nearly neutral, making it distinct from counties with larger margins. Such environments maximize the likelihood of exposure to both political viewpoints. For our analysis, this separation is particularly important because it sharpens contrasts in mobility patterns and strengthens the interpretation of observed differences. Distinct travel patterns between red, blue, and purple counties can thus be attributed more confidently to political factors. If the threshold were much higher, “purple” would include moderately leaning areas, blurring the lines between categories.

Traveling to politically similar counties implies that the origin and destination share the same ideological leaning, such as traveling from one blue county to another blue county. Conversely, traveling to politically opposing counties indicates that the origin and destination have differing ideological leanings, such as traveling from a blue county to a red county, or vice versa. The travel polarization index was calculated as the difference between trips to politically similar counties and trips to politically opposing counties, divided by the total number of trips. The formula is as follows:


$$I=\;\frac{T_{s}-\;T_{o}}{T_{s}+\;T_{o}+\;T_{P}}$$

Where I denotes the traveling polarization index, $T_{s}$ represents the number of trips to politically similar counties, $T_{o\;}$ represents the number of trips to politically opposing counties and $T_{P}$ indicates the number of trips to purple counties. The values of I range from −1–1, with 0 indicating a balance in trips to both types of destinations. Values closer to 1 indicate a greater likelihood of traveling to politically similar counties, while values closer to −1 suggest a greater likelihood of traveling to politically opposing counties. Our measurement accounted for the total number of trips, including those to purple counties, in order to avoid selection bias and enhance validity. The traveling polarization index was calculated only for blue and red origin counties, as purple counties did not have politically opposing counterparts.

#### Control variables.

We controlled for several demographic characteristics at the county level. Income was measured as median household income; gender ratio as the percentage of males in the population; white ratio as the percentage of residents identifying as white; and education ratio as the percentage of residents with a bachelor’s degree or higher. All demographic data were obtained from the U.S. Census Bureau.

In addition, travel appeal and economic opportunity capture the inherent attractiveness of county destinations, while travel distance denotes the estimated geographical distance between origin and destination counties. These variables were obtained from datasets provided by the U.S. Department of Agriculture and the National Bureau of Economic Research. State political leaning refers to the political leaning of the state in which an origin county is located, measured as the difference between the percentage of Republican and Democratic votes in the 2020 general election. Finally, purple travel share is defined as the percentage of trips directed to purple counties.

Table 1 presents the descriptive statistics for the key variables and controls, including their means, standard deviations, and ranges.

**Table 1:** Descriptive statistics for key variables and controls.

Variables	Mean	SD	Ranges
Traveling polarization index	0.17	0.85	[−1, 1]
Political leaning	−0.24	0.31	[−0.88, 0.50]
Political leaning strength	0.32	0.22	[0.03, 0.87]
Household median income	0.30	0.20	[0, 1]
Gender ratio	0.49	0.01	[0.47, 0.52]
White ratio	0.66	0.15	[0.26, 0.90]
College attainment	0.26	0.07	[0.13, 0.48]
Travel appeal	0.04	0.19	[0, 1]
Economic opportunity	0.10	0.13	[0, 1]
Travel distance	0.28	0.20	[0, 1]
State political leaning	−0.02	0.21	[−0.88, 0.33]
Purple travel share	0.07	0.20	[0, 0.91]

### Multicollinearity test

**Fig 1:**
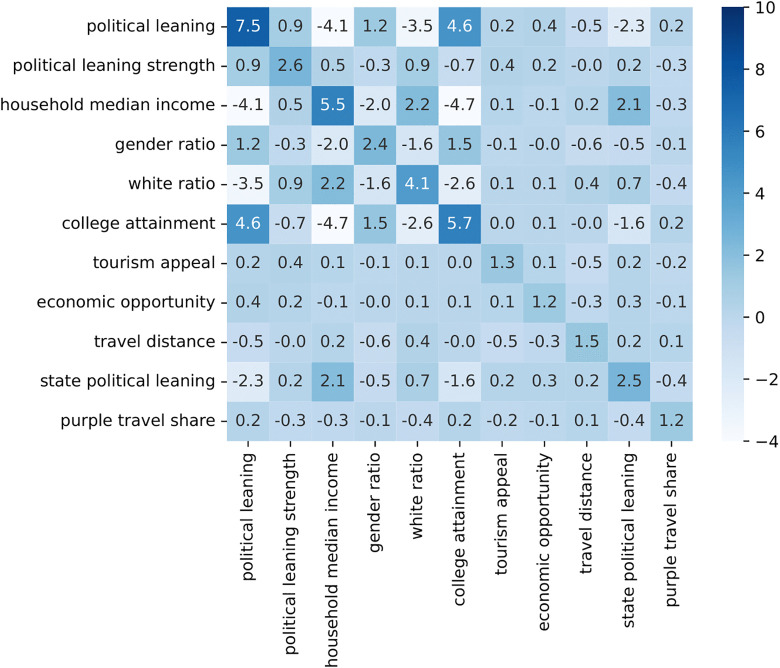
Variance inflation factors for independent variables and controls.

To assess multicollinearity, we calculated Variance Inflation Factors (VIFs) for all independent variables and controls. As shown in Fig 1, the VIF values for all variables were below the commonly accepted threshold of 10, indicating that the variables were not highly correlated. Accordingly, no variables were excluded from the regression model. This diagnostic supports the validity of our regression results.

A key strength of this study lies in the integration of electoral and mobility data. By linking county-level presidential election returns with large-scale phone-based travel records, we are able to examine how partisan geography intersects with everyday movement, thereby extending research on geographical polarization beyond residential neighborhoods into broader activity spaces. This novel combination of datasets also enables the development of the traveling polarization index, an innovative outcome measure that captures mobility preferences for politically similar destinations. In doing so, the study provides new insights into partisan identity and geographical sorting by demonstrating how political context shapes patterns of movement through social and public space.

Our findings should be interpreted at the county level rather than the individual level, as extending them to individual travel behavior risks an ecological fallacy—making inferences about individuals based on aggregate data. This limitation stems from the lack of individual-level travel data, which data companies rarely share due to privacy concerns. Instead, we rely on tract-to-tract mobility flows and analyze collective patterns, a less intrusive approach that has become increasingly common in mobility research [12,38]. A further limitation is that our dataset, while derived from more than 40 million smartphones, is not fully representative of the U.S. population, with greater coverage in metropolitan areas than in rural regions. These constraints limit the generalizability of our findings.

## Results

**Fig 2:**
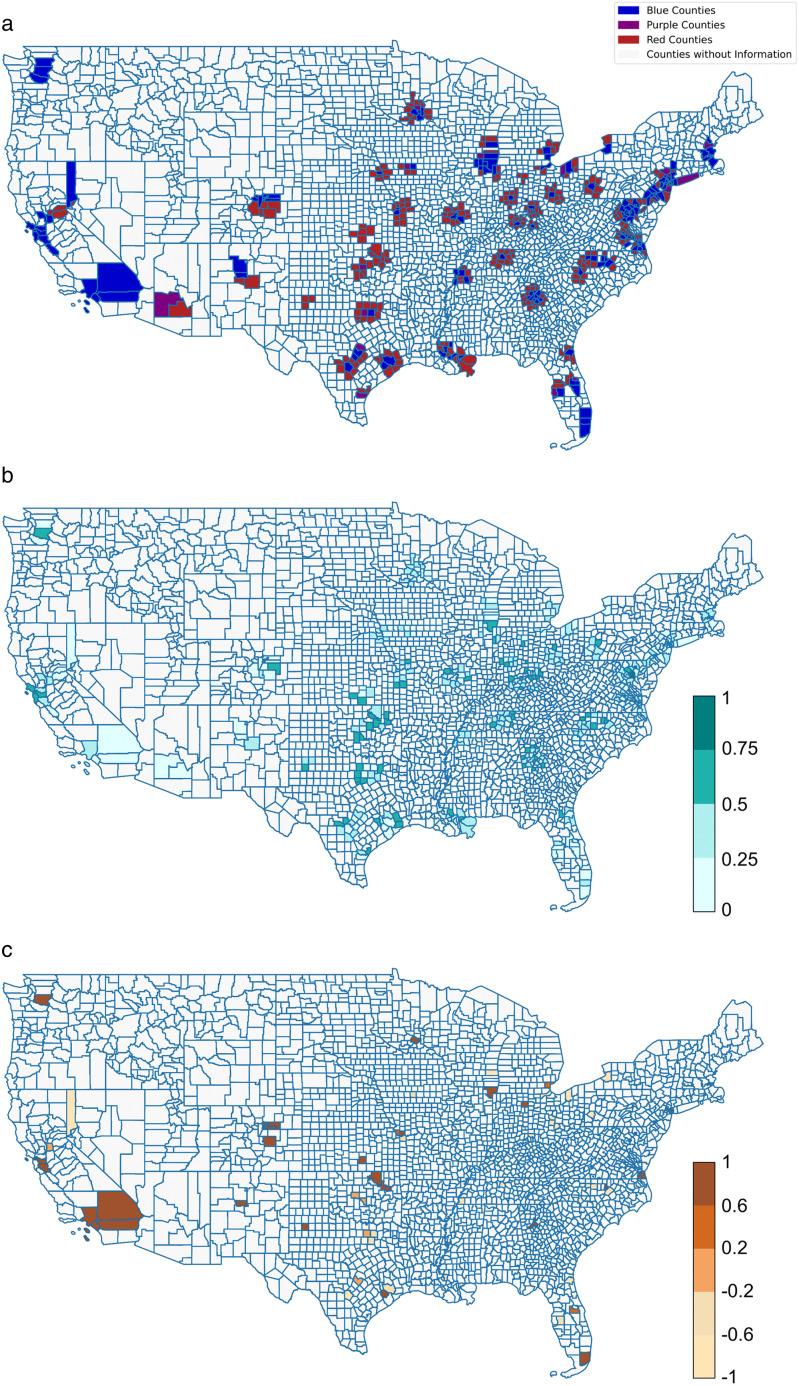
Mapping political leaning, political leaning strength, and traveling polarization index of counties [34]. a. Political leaning of counties as origins and destinations. b. Political leaning strength of counties as origins and destinations. c. Traveling polarization index of origin counties.

Fig 2 visualizes political leanings, political leaning strength, and the traveling polarization index on a map of U.S. counties. Specifically, Fig 2a illustrates the political leanings of the 471 counties in our dataset. As shown, these counties are geographically diverse, spanning 37 states across the mainland United States. The ideological composition of these counties is relatively balanced, with 162 blue counties, 17 purple counties, and 292 red counties. Fig 2b illustrates the political leaning strength across these counties. In 88 counties, the political leaning strength exceeds 0.5, indicating strong political alignment. These counties with solid political leanings are distributed across many states nationwide. In addition, Fig 2c presents the traveling polarization index for 83 blue and red counties as origins. Among these, 49 counties have a traveling polarization index greater than 0, reflecting a tendency to travel to politically similar locations.

**Fig 3:**
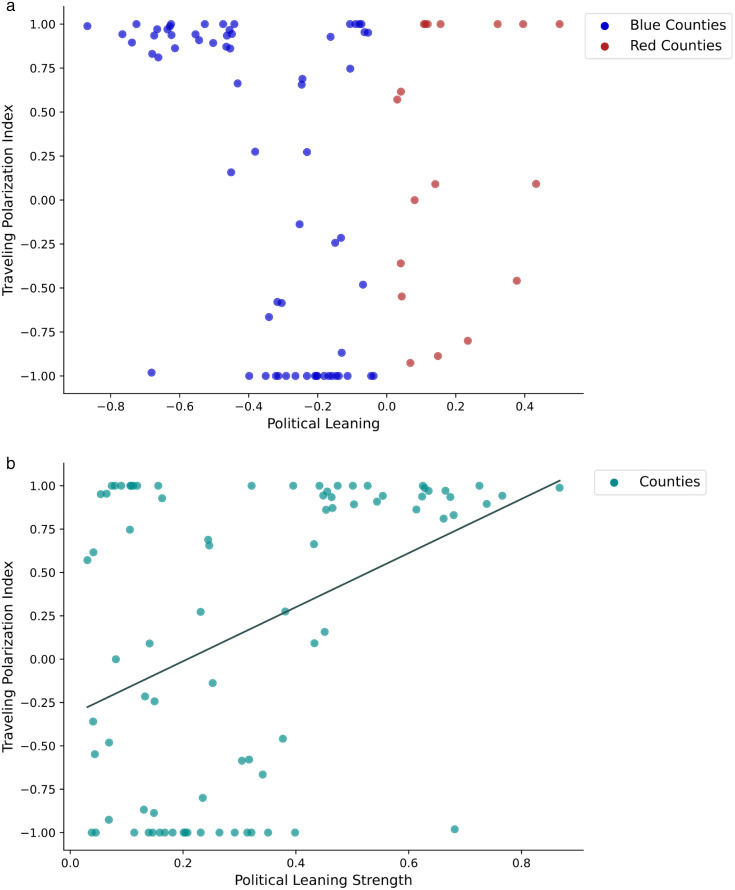
a. The relationship between political leaning and traveling polarization index. b. The relationship between political leaning strength and traveling polarization index.

H1 predicts that political leaning is associated with mobility preferences for traveling to politically similar locations. Specifically, individuals traveling from conservative counties are likely to visit politically congruent destinations than those traveling from liberal counties. To test this hypothesis, we conducted an OLS regression analysis of county-to-county movements. This analysis assessed the effect of political leaning on the traveling polarization index, enabling us to compare mobility preferences between travelers from conservative and liberal counties. Control variables included county demographics, tourism appeal, economic opportunity, purple travel share, and state political leaning. The relationship between political leaning and the traveling polarization index is illustrated in Fig 3a. As reported in Table 2, political leaning is not significantly associated with the traveling polarization index (*B *= 0.69, *t *= .98, *p *= .33). Thus, H1 is not supported. In other words, we do not find evidence to support that political leaning shapes mobility preferences nor that U.S. counties are divided along partisan lines in terms of geographical mobility.

**Table 2:** OLS regressions between political leaning, political leaning strength, and traveling polarization index.

	Traveling polarization index	
	β	SE
Intercept	−13.71*	5.69
Political leaning	0.69	0.71
Political leaning strength	2.12***	0.57
Covariates		
Household median income	−0.04	0.95
Gender ratio	29.09*	12.35
White ratio	−0.73	1.01
College attainment	−1.39	2.62
Economic opportunity	1.16	0.73
Tourism appeal	0.20	0.32
Travel distance	−0.27	0.45
Purple travel share	−0.22	0.42
State political leaning	−1.07	0.72
Adjusted R^2^	0.32	
F	(11, 71) = 4.44	

*p < .05, **p < .01, ***p < .001.

H2 posits that political leaning strength is positively associated with mobility preference for traveling to politically similar locations. The OLS regression analysis enables us to test whether individuals traveling from counties withstrong or weak political leanings are more likely to visit politically congruent areas. The relationship between the two variables is presented in Fig 3b. As shown in Table 2, political leaning strength has a significant impact on the traveling polarization index (*B* = 2.12, *t* = 3.74, *p* < .001), supporting H2. Individuals traveling from counties with strong political leanings tend to make trips to politically similar destinations. The more pronounced the political leaning of an origin county, the stronger the mobility preference for politically congruent destinations is.

**Fig 4:**
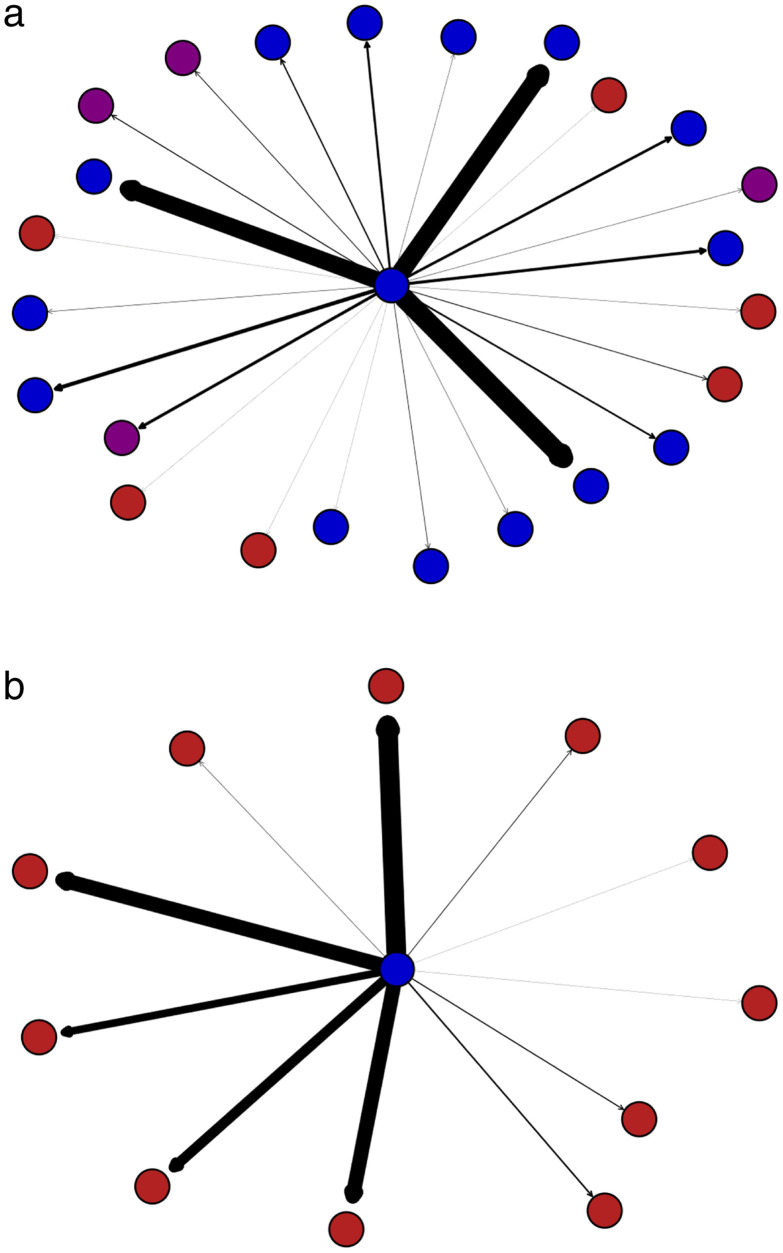
a. Travel network for New York County with a high traveling polarization index and strong political leaning. b. Travel network for Jefferson County in Kentucky with a low traveling polarization index and weak political leaning.

Fig 4 utilizes travel networks to illustrate the relationship between political leaning strength and mobility preference. Each node represents a county, with distinct colors indicating its ideological leaning, consistent with the color scheme used in previous graphs. An edge is added between two nodes if at least one trip was made between them. The connections are directional, represented by arrows: the start of an arrow indicates the origin, and the end of an arrow indicates the destination. The edge weight corresponds to the number of trips made between two nodes. The greater the number of trips, the wider the edge appears. As shown in Fig 4a, New York County has a strong political (liberal) leaning, and those traveling from this county make substantially more trips to politically aligned areas. By contrast, Fig 4b illustrates that Jefferson County, which has a weak liberal leaning, records a higher number of trips to politically opposing counties.

### Robustness checks

There is no universally agreed-upon threshold in election results for classifying county-level partisan leanings. Prior research and political analysis have employed various cutoffs, including ±3% [39], ± 5% [40], and ±10% [41,42]. Political leaning exists on a continuum; whether one picks 3%, 5%, or 10% to delineate categories will affect how many counties fall into each bucket and potentially influence analytical results. We acknowledge that a ± 3% threshold is relatively stringent and could overstate the number of counties labeled as firmly partisan. To address this limitation, we conducted robustness checks with higher thresholds. Specifically, we re-ran our key analyses using alternative cutoffs of ±5% and ±10% to classify blue, purple, and red counties. The robustness checks confirm that our findings remain consistent across alternative thresholds. Regression analyses using different cutoff points yield a similar pattern: political leaning is not significantly associated with mobility preference, whereas political leaning strength is. These results indicate that our conclusions are robust and not sensitive to the specific threshold employed.

## Discussion and conclusions

We investigate the impact of political leaning and political leaning strength on mobility preferences for politically similar locations. The effects of political polarization have spilled over into residential areas, with partisans increasingly clustering in neighborhoods alongside like-minded individuals [3].Our study seeks to explore whether individuals further reinforce geographical sorting by traveling to politically similar destinations. Through analyzing human mobility patterns between counties, we find that political leaning is not significantly associated with mobility preferences for politically similar locations. Simply put, we do not find evidence that travel preferences align with political leaning or that U.S. counties are divided in geographical mobility across partisan lines.

This null result appears inconsistent with previous findings. Prior research has shown that political identity can be a significant factor influencing individuals’ mobility decisions [12,21].On the one hand, individuals may make travel decisions that favor political in-group members [23]. On the other hand, people may avoid traveling to certain places to minimize partisan conflict [22]. However, in our study, we do not observe an alignment between political leaning and mobility preferences for traveling to politically congruent locations. That is to say, it is possible for an individual to travel from liberal areas to conservative areas, and vice versa. Political polarization may have spread to residential neighborhoods [15], but communication between politically different districts is still likely to occur, at least for districts with moderate or weak political leanings.

Furthermore, our analysis does not support the hypothesis that individuals from conservative areas show a stronger tendency toward politically congruent travel. Although prior research suggests that Republicans often express more intense outgroup avoidance and ingroup favoritism than Democrats [27], such partisan affect may not play a decisive role in everyday mobility decisions. Instead, individuals may prioritize practical considerations—such as convenience, cost, or amenities—over political alignment when choosing destinations [12,43]. Thus, mobility may reflect shared functional needs rather than ideological sorting. These possibilities remain speculative, however, and future research should examine the specific factors that drive mobility preferences and clarify under what conditions political contexts may influence travel choices.

In addition, we discover that political leaning strength is positively associated with mobility preferences for traveling to politically similar locations. In particular, individuals traveling from counties with strong political leanings are more likely to visit politically congruent areas, regardless of whether the origin county is liberal or conservative. Conceptually, this pattern indicates that political leaning strength functions as a salient social identity marker that structures spatial practices in the dynamic domains, consistent with the predictions of SIT. The theory holds that individuals tend to enact ingroup favoritism and outgroup avoidance when stable identities are present; our results show these identity processes extend into geographical patterns of mobility, shaping where people choose to go.

This identity-based interpretation is also consistent with prior research showing that polarization is most pronounced among strong partisans [29]. Partisans tend to display warmer evaluations of ingroup members and colder evaluations of outgroup members [11]. We contribute by demonstrating a behavioral manifestation of these affective patterns: travelers from strongly partisan contexts select destinations that affirm rather than challenge their political identity. In other words, mobility may become a site for identity maintenance. This finding complements the literature on residential sorting, which emphasizes long-term locational choice, by documenting short-term, dynamic sorting in people’s activity spaces. Together, these patterns imply that partisan identity likely structures both where people live and where they move day to day.

In addition, this study advances our understanding of geographical polarization. The results suggest that partisan clustering is not confined to residential neighborhoods; it also propagates through movement, shrinking opportunities for cross-partisan contact even when people are outside their home counties. The study reveals more segregated mobility flows especially in strongly partisan counties, underscoring travel as a new domain in which political identities shape social and spatial interactions.

This study has implications for future research and policy design. First, it highlights a new research avenue for examining the intersection of geographical polarization and human mobility. Building on our findings, future work should investigate the processes and political consequences of dynamic spatial segregation. For instance, when individuals travel to politically opposing counties, what forms of cross-cutting interactions may occur, and how might these influence voting behavior? Second, future studies should employ more fine-grained and representative mobility data. Access to individual-level records would allow researchers to better identify the mechanisms through which political leaning shapes mobility preferences, providing more nuanced insights into the politics of human movement. Although large-scale datasets capture extensive travel patterns, they do not necessarily reflect the broader population. Using more representative data would enhance the generalizability and robustness of findings. Finally, our results provide guidance for designing contextual interventions to reduce political polarization. Intergroup contact is recognized as a promising strategy for alleviating partisan divisions [44,45]. Our study suggests that such contact, facilitated through travel, is more likely in politically competitive areas than in homogeneous ones. Interventions that foster cross-cutting engagement—such as community events or dialogue initiatives—may therefore be most effective when targeted in politically mixed contexts. Implementing such strategies in these contexts could help increase opportunities for intergroup contact and thereby contribute to mitigating polarization.

### Notes

In this paper, we address geographical mobility simply as mobility.

## Supporting information

S1 Fig Ideological composition of origin and destination counties. (DOCX)
