# Supplementary material for: Political polarization on the move: Analyzing geographical mobility between counties in the U.S
Source: PLoS One. 2026 Jan 28;21(1):e0339333. doi: 10.1371/journal.pone.0339333 (PMC12851469; doi:10.1371/journal.pone.0339333)
Supplement: S1 Fig — (DOCX) [file pone.0339333.s001.docx]

a.


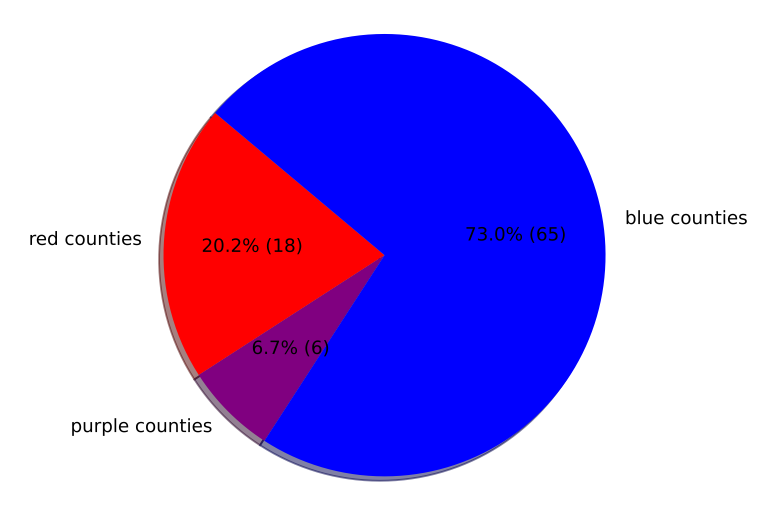


b.


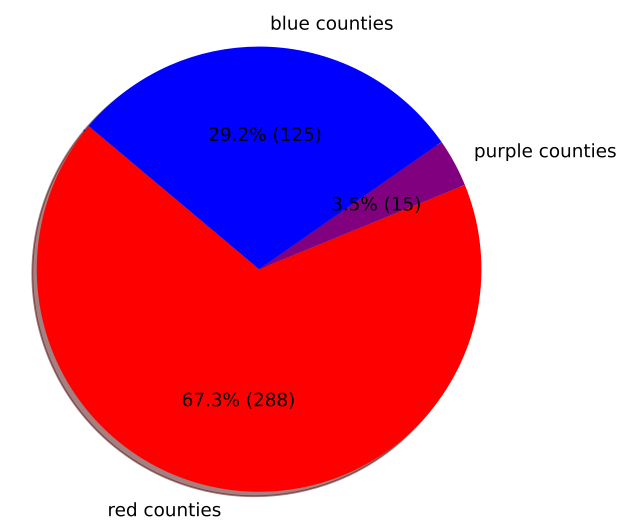


**Fig 5. Ideological composition of origin and destination counties**

Fig 5 illustrates the ideological composition of both origin and destination counties. Among origins, 73% are classified as liberal and about 20% as conservative. By contrast, the composition of destinations is reversed: 67.3% are red counties, while 29.2% are blue counties.
